# Supplementary material for: Quadrature squeezing in a nanophotonic microresonator
Source: Nat Commun. 2025 Nov 28;16:10791. doi: 10.1038/s41467-025-66703-x (PMC12663586; doi:10.1038/s41467-025-66703-x)
Supplement: Supplementary file 1 — Supplementary Information [file 41467_2025_66703_MOESM1_ESM.pdf]

Supplementary Information - Quadrature squeezing in a nanophotonic microresonator

Alexander E. Ulanov[1,†], Bastian Ruhnke[1], Thibault Wildi[1], Tobias Herr[1,2,*]

[1]Deutsches Elektronen-Synchrotron DESY, Notkestr. 85, 22607 Hamburg, Germany
[2]Physics Department, University of Hamburg UHH, Luruper Chaussee 149, 22761 Hamburg, Germany
[†]alexander.ulanov@desy.de
[*]tobias.herr@desy.de

1 Dual-pump preparation

To generate the dual-pump with approximately 600 GHz line separation, we use an electro-optic comb (EOC) generator consisting of two cascaded electro-optic phase modulators. The modulation signal is derived from a frequency tunable microwave source that is amplified and adjusted in phase before being coupled to the modulators. A continuous-wave master laser passes through this setup, producing an optical frequency comb with a line spacing defined by the modulation frequency of ≈ 16.67 GHz, and spanning approximately 733 GHz (44 sidebands).

The EOC is sent to a programmable optical filter (WaveShaper), which is configured to transmit only ~ 20 GHz spectral windows centered on the ±18th sidebands of the EOC. This effectively generates two optical sidebands symmetrically centered around the original master laser frequency. These two sidebands are then amplified using an erbium-doped fiber amplifier (EDFA). Amplified spontaneous emission (ASE) at the master laser wavelength is suppressed via a fiber-based Bragg grating in transmission, before coupling the dual-pump into the photonic chip.

2 Balanced homodyne detection

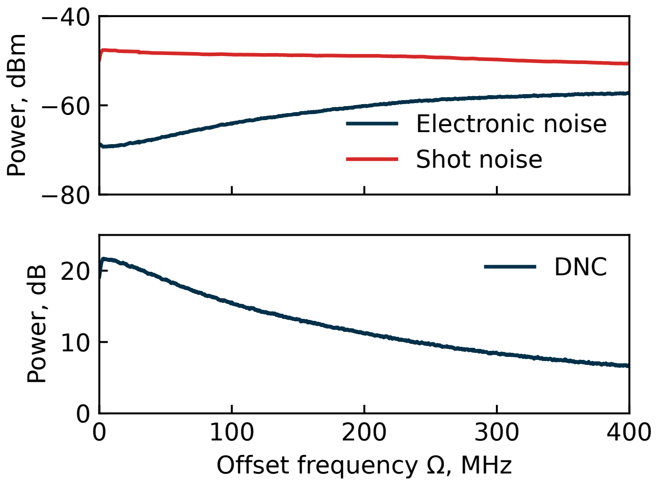

Figure S1 | Top: Electronic (blue) and shot noise (red) spectra of the balanced homodyne detector. Bottom: Detector dark noise clearance (DNC).

After spectral filtering (cf. main text), the generated squeezed light is overlapped with the local oscillator (LO) derived from the continuous-wave master laser, and directed to a free-space balanced homodyne detector (BHD). To characterize the BHD dark noise clearance (DNC), we measure its electronic noise (with the local oscillator blocked) and shot noise (with the local oscillator unblocked). The DNC is also used to characterize the frequency dependence of the BHD detection.

The output of the BHD is connected to an electronic spectrum analyzer. Measurements are taken with a resolution bandwidth of 2 MHz, a video bandwidth of 100 Hz, and LO power of 16 mW. The same settings are used to measure squeezing. The spectra of both measurements, as well as the extracted DNC, are presented in Fig. S1. In our setup, the DNC exceeds 20 dB at low frequency offsets Ω and gradually decreases with higher offset frequency. The quantum efficiency of the photodetectors is ≈ 0.75.

3 Impact of unsuppressed parasitic nonlinear processes

Based on the formalism presented in the Methods section of the main text (the same approach used to generate Fig. 2a), we perform additional numerical simulations to quantify the impact of parasitic nonlinear processes for different normalized parasitic mode splittings, β. The results are shown in Fig. S2, where the blue dots indicate the level of squeezing at zero frequency offset ($\Omega = 0$). Generally, larger β lead to improved squeezing, however, there is a saturation with larger β, given by the deterministic bound (black dashed line) that assumes complete absence of parasitic processes and an outcoupling efficiency of $\eta = 0.9$. The red dashed line shows the deterministic bound for $\eta = 0.886$. By comparison, one can infer that, under our experimental conditions ($\beta \approx 13.5$ and $\eta = 0.9$), our system performs effectively as a system with $\eta = 0.886$ at the deterministic bound (i.e. without parasitic processes). Thus, we can estimate that the contribution of unsuppressed parasitic nonlinear processes corresponds to an effective loss of only approximately $1 - 0.886/0.9 \approx 1.6\%$.

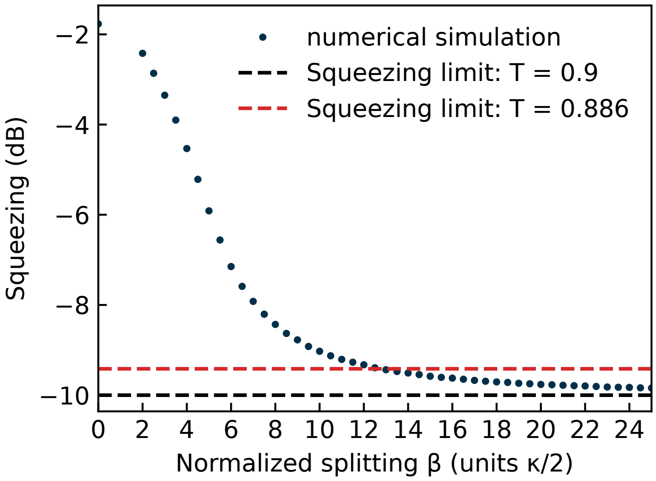

Figure S2 | Numerically simulated squeezing at zero frequency offset (blue dots) for different normalized mode splitting β and theoretical squeezing levels corresponding to quantum efficiencies of 0.9 and 0.886 (black and red dashed lines, respectively).

4 Dependence of total efficiency T and squeezing parameter r on pump power

We use the theoretical relation presented in the *Effect of losses* Methods section of the main text with the experimentally measured squeezing and anti-squeezing levels as a function of total pump power (see Fig. 3c in the Main text), to extract the dependence of the total efficiency T and the squeezing parameter r on the total on-chip pump power [1, 2]. The results are presented in Fig. S3. We observe that the squeezing parameter r grows linearly with the pump power, as expected from the Kerr Hamiltonian, while the total efficiency T, remains nearly unchanged.

5 Degenerate optical parametric oscillation threshold

We measure the generated degenerate optical parametric oscillation (DOPO) power as a function of the on-chip pump power. These measurements are done using a high-sensitivity optical spectrum analyzer operating in zero-span mode, centered at the signal wavelength. The resulting data (see Fig. S4) follows the exponential dependence predicted by the dual-pump DOPO theory [3]. From this data, we extract a DOPO threshold of 39.36 ± 2.19 mW, where the uncertainty stems from the measurement uncertainties of both the pump power and the DOPO power.

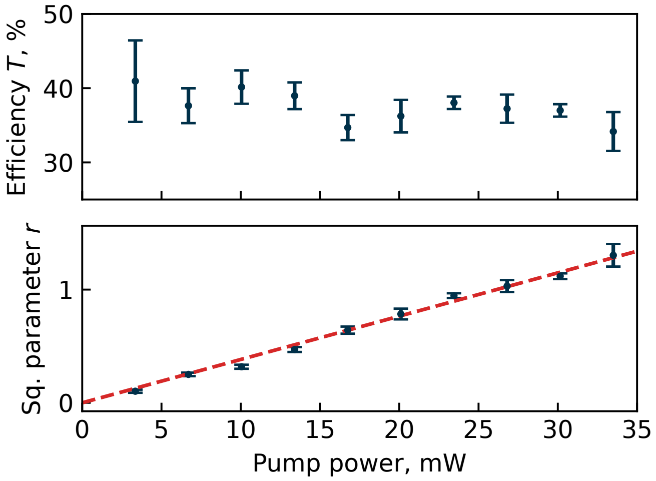

Figure S3 | Dependence of the total efficiency, T (top), and the squeezing parameter, r (bottom), on the total pump power. Error bars represent the 1σ confidence intervals.

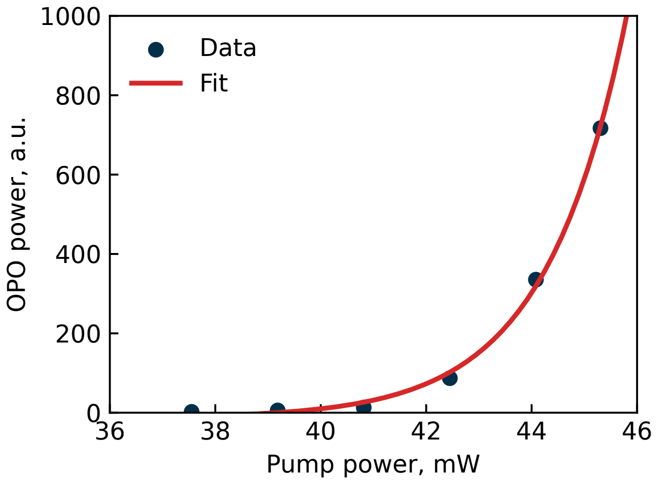

Figure S4 | Experimentally measured generated DOPO power as a function of on-chip pump power (blue dots), along with an exponential fit (red line).

References

1. Scully, M. O. & Zubairy, M. S. *Quantum Optics* (Cambridge University Press, Cambridge, 1997). 55
2. Lvovsky, A. I. in *Photonics: Scientific Foundations, Technology and Applications* 121–163 (John Wiley & Sons, Ltd, 2015).
3. Okawachi, Y. *et al.* Dual-Pumped Degenerate Kerr Oscillator in a Silicon Nitride Microresonator. *Optics Letters* **40,** 5267 (2015).
